# Latent HIV-1 can be reactivated by superinfection in a Tat-dependent manner, which can lead to the emergence of recombinant viruses

**DOI:** 10.1186/1742-4690-10-S1-P27

**Published:** 2013-09-19

**Authors:** Daniel Aaron Donahue, Sophie M Bastarache, Richard D Sloan, Mark A Wainberg

**Affiliations:** 1 McGill University AIDS Centre, Montreal, QC, Canada; 2 Centre for Immunology and Infectious Disease, Blizard Institute, Barts and The London School of Medicine and Dentistry, Queen Mary University of London, London, UK

## Background

The HIV-1 latent reservoir represents an important source of genetic diversity that could contribute to viral evolution and drug resistance. Latent virus reactivation might occur by superinfection of latently infected cells. Previous studies have suggested that latent viruses contribute to recombination *in vivo*, but this has not been experimentally studied.

## Methods

We used both Jurkat and primary cell latency models. In Jurkat cells, wt and drug-resistant latent populations were superinfected with wt or drug-resistant viruses, including nef or tat mutants, and treated with various inhibitors. Viral reporter gene expression was measured by FACS. Sequence tags on each resistant virus permitted identification of recombinants. We also performed superinfection experiments in primary cells.

## Results

Latent viruses were reactivated by superinfection in both latency models. In Jurkat cells, the extent of latent virus reactivation was strongly correlated (r^2^ = 0.98) with the extent of superinfection across a wide range of viral inocula. Latent virus reactivation required gene expression of the superinfecting virus, since latent viruses were reactivated in the presence of a protease inhibitor, but not a reverse transcriptase (RT) or integrase inhibitor. Latent virus reactivation occurred following superinfection with nef-deleted or tat-attenuated, but not tat-inactivated, viruses. These results suggest that neither gp120-induced CD4/CXCR4 signalling nor Nef-induced NFkB/NFAT modulation were required for latent virus reactivation, but that superinfecting virus Tat was required. Drug-resistant latent viruses (RT K103N) were reactivated following superinfection with additional drug resistant viruses (RT M184V). The resulting supernatants (containing heterozygous virions) were used to infect new cells, to which the RT inhibitors FTC and EFV were added. Sequencing and restriction digestion confirmed that recombination frequently occurred, leading to FTC/EFV-resistant virions encoding unique sequence tags derived from both parental viruses. We also established latency in unstimulated primary CD4T-cells and subjected them to superinfection. Results from nine individual donors indicate that latent viruses were reactivated by superinfection; these results were statistically significant (p=0.001).

## Conclusions

Our results suggest that superinfection of latently infected cells can reactivate latent viruses, which can then recombine with the superinfecting virus. Since all viral quasispecies including drug-resistant viruses can be latently archived, reactivation of latent viruses by superinfection or other means could contribute to the emergence of replicatively fit viruses in the face of strong selective pressures.

